# High Resolution, High Contrast Beamformer Using Minimum Variance and Plane Wave Nonlinear Compounding with Low Complexity

**DOI:** 10.3390/s21020394

**Published:** 2021-01-08

**Authors:** Xin Yan, Yanxing Qi, Yinmeng Wang, Yuanyuan Wang

**Affiliations:** 1 Department of Electronic Engineering, Fudan University, Shanghai 200433, China; 19110720035@fudan.edu.cn (X.Y.); 17110720012@fudan.edu.cn (Y.Q.); 18210720044@fudan.edu.cn (Y.W.); 2 Key Laboratory of Medical Imaging Computing and Computer Assisted Intervention of Shanghai, Shanghai 200032, China

**Keywords:** plane wave compounding, minimum variance, generalized sidelobe canceler, dimension reduction, delay multiply and sum, nonlinear compounding, low complexity

## Abstract

The plane wave compounding (PWC) is a promising modality to improve the imaging quality and maintain the high frame rate for ultrafast ultrasound imaging. In this paper, a novel beamforming method is proposed to achieve higher resolution and contrast with low complexity. A minimum variance (MV) weight calculated by the partial generalized sidelobe canceler is adopted to beamform the receiving array signals. The dimension reduction technique is introduced to project the data into lower dimensional space, which also contributes to a large subarray length. Estimation of multi-wave receiving covariance matrix is performed and then utilized to determine only one weight. Afterwards, a fast second-order reformulation of the delay multiply and sum (DMAS) is developed as nonlinear compounding to composite the beamforming output of multiple transmissions. Simulations, phantom, in vivo, and robustness experiments were carried out to evaluate the performance of the proposed method. Compared with the delay and sum (DAS) beamformer, the proposed method achieved 86.3% narrower main lobe width and 112% higher contrast ratio in simulations. The robustness to the channel noise of the proposed method is effectively enhanced at the same time. Furthermore, it maintains a linear computational complexity, which means that it has the potential to be implemented for real-time response.

## 1. Introduction

Ultrasound imaging has been widely used in medical clinical diagnosis due to the advantages of non-invasive, high efficiency and low cost. The traditional focus scanning mode makes the imaging result of rapid-moving tissues and organs blurred because of the low frame rate. This affects the diagnostic accuracy of clinicians. Ultrafast imaging mode is able to overcome such problems since it can provide a high frame rate (thousands of frames per second). Thus, it is more advantageous in the applications of 3D and 4D ultrasound imaging, elastography, doppler blood flow imaging, and so on [1,2,3]. The plane wave compounding (PWC) is an effective way to realize ultrafast ultrasound imaging, which was originally proposed by Lu and Cheng [4,5], and then further improved by Montaldo et al. [6]. By combining low-resolution imaging results from different transmitting steering angles, a high-resolution imaging result can be generated. More transmitting beams can generate higher imaging quality, while the frame rate will be decreased accordingly, and vice versa. Therefore, how to achieve the better imaging performance while maintaining the high frame rate of the PWC is of great significance for the further applications of the ultrafast ultrasound imaging in the medical ultrasound imaging field.

Better imaging performance means better imaging resolution and better imaging contrast. As is known, the minimum variance (MV) method proposed by Capon in 1969, achieved a certain effect on improving the imaging resolution, while the imaging contrast was still unsatisfactory [7]. Several studies have focused on modifying the MV method and applying it to PWC imaging. Zhao et al. proposed the joint transmitting-receiving (JTR) MV beamformer, in which two MV weights are calculated to obtain the improvement of the resolution [8]. A mixed transmitting-receiving (MTR) proposed by Wang et al. further enhanced the imaging quality by redefining the MV optimization problem [9]. Nguyen et al. proposed a spatial coherence approach to implement the MV beamformer using data-compounded methods [10]. Other methods combining the MV with the coherence factor (CF) were also widely studied to further improve the beamforming performance [11,12,13]. However, the calculation complexity of above MV-based methods is high mainly due to the estimation and inversion of the covariance matrix. This limits the application of these beamformers in real-time imaging systems.

To alleviate the computational load of the MV, several studies have been proposed. Asl et al. introduced the Toeplitz structured covariance matrix to reduce the calculation amount [14]. Beam-space methods have also been proposed to project received signals to a lower dimensional space and the MV is then implemented on the new space [15,16]. Another low complexity MV beamformer implemented with a partial generalized sidelobe canceler (GSC) for phased array imaging was proposed by Deylami et al. in 2019. The partial GSC divided the weight vector into one constant and one adaptive weight, so that the optimization process could be implemented with lower complexity on the adaptive part [17]. Although these methods reduce the calculation amount, their imaging quality still needs to be improved for the PWC.

The delay multiply and sum (DMAS) is an effective method to improve the imaging contrast, which was first introduced to the ultrasound field by Matrone et al. in 2015 [18]. The spatial coherence of signals was introduced to the beamforming process and the sidelobe clutter was suppressed owing to the low coherence relative to the desired signal. However, the imaging resolution and computational complexity still could not meet requirements for the PWC. Several improvement strategies for the DMAS are proposed in recent years [19,20,21]. Mozaffarzadeh et al. proposed a double-stage DMAS to further increase the contrast by using the DMAS to replace the delay and sum (DAS) algebra in the expansion formula. However, the burden of multiplications is even heavier with this approach [22], which limits its further applications. In addition, a real-time implementation of the DMAS has been used for calculating the amount of reduction with no negative effect on the imaging result [23,24]. The DMAS used as nonlinear compounding, named as the frame multiply and sum (FMAS), was also introduced to improve the contrast for PWC imaging [25]. Nevertheless, there is still room for the improvement in the imaging resolution and contrast while retaining the real-time ability.

In this paper, we tend to propose a beamforming scheme to improve the imaging quality for the PWC. A cascade structure including adaptive weighting and nonlinear compounding stages is proposed. In stage 1, a MV weighting vector of the receiving array dimension is calculated to obtain high resolution images through the partial GSC structure [17]. We name it the pGSC for short in the following sections. The specified submatrix spatial smoothing process is implemented and the multi-wave covariance matrix is calculated by averaging each covariance result of different transmissions [8]. Afterwards, the blocking and dimension reduction matrices are selected to determine the weighting vector, and the equation expansion step is also adopted to further alleviate the computational burden. The following stage is a nonlinear compounding procedure, which is mainly designed for the higher imaging contrast. An efficient second-order signed delay multiply and sum (2-sDMAS) method is proposed to compound the beamforming result of each transmitting event. Then, it is referred to as second-order signed frame multiply and sum (2-sFMAS). A multiply and sum (MAS) process is used to replace the simple DAS algebra in the real-time implementation equation of the DMAS. Afterwards, the sign of the traditional DAS result is adopted to make a correction to the previous compounding result. Through two stages, a higher imaging quality can be obtained with a fast imaging speed.

The rest of this paper is arranged as follows. Background of related beamforming methods is presented in Section 2. The specific implementation of the proposed method is illustrated in Section 3. Section 4 shows the setup, data acquisition, evaluation metrics and results of experiments. In Section 5, further discussions and explanations are given. At last, a conclusion is made in Section 6.

## 2. Backgrounds

### 2.1. Data Model of Plane Wave Compounding

The PWC is a process of recombining plane wave imaging results from different transmissions. Here, we assume that a transducer with *M* elements is used to transmit *N* plane waves. Then, the echo signal is recorded into a 2-D data matrix **X**(*n*):

(1) $$X(n)=\left[\begin{array}{cccc} x_{1,1}(n) & x_{1,2}(n) & \cdots & x_{1,M}(n)\\ x_{2,1}(n) & x_{2,2}(n) & \cdots & x_{2,M}(n)\\ \vdots & \vdots & \ddots & \vdots \\ x_{N,1}(n) & x_{N,2}(n) & \cdots & x_{N,M}(n) \end{array}\right]=\left[\begin{array}{c} x_{1}[n]\\ x_{2}[n]\\ \vdots \\ x_{N}[n] \end{array}\right],$$

where *x_i_*,_*j*_(*n*) represents the echo data from the *i*th transmitting event received by the *j*th element and $x_{i}[n]$ is the corresponding data vector of the *i*th transmission. The term *n* is the time index.

By using the traditional DAS method, we can get the output of coherent plane wave compounding. The mathematical expression is:

(2) $$y_{DAS}=\frac{1}{MN}\sum_{i=1}^{N}\sum_{j=1}^{M}x_{i,j}(n).$$

### 2.2. Delay Multiply and Sum Beamformer

The DMAS beamformer suppresses uncorrelated signals by calculating the spatial cross-correlation of received array signals [18]. For a single transmission, received array signals can be expressed as:

(3) $$x(n)={\left[x_{1}(n),x_{2}(n),\cdots x_{M}(n)\right]}^{H}.$$

where ${\left(\cdot \right)}^{H}$ represents the matrix conjugate transpose.

Then the beamforming output of the DMAS is calculated as:

(4) $$y_{DMAS}(n)=\sum_{j=1}^{M-1}\sum_{l=j+1}^{M}sign(x_{j}(n)x_{l}(n))\cdot \sqrt{\left|x_{j}(n)x_{l}(n)\right|}*f,$$

where *x_j_*(*n*) and *x_l_*(*n*) are delayed signals received by the element *j* and element *l*, respectively. *M* is the number of transducer elements. *f* denotes a bandpass (BP) filter, which aims to attenuate the direct current (DC) and higher frequency components while preserving the second harmonic component generated by the multiplication process [18].

In addition, the implementation of the DMAS was further optimized [23,24]. Each echo data was rescaled by applying a signed square root operation. It is expressed as:

(5) $$\bar{x_{j}}(n)=sign(x_{j}(n))\cdot \sqrt{\left|x_{j}(n)\right|}.$$

Then, the beamforming output expression of the DMAS is changed to the following equation:

(6) $$y_{DMAS}(n)=\sum_{j=1}^{M-1}\sum_{l=j+1}^{M}\bar{x_{j}}(n)\cdot \bar{x_{l}}(n)*f.$$

Based on the Equation (6), a simplified implementation form is expressed as follows:

(7) $$y_{DMAS}(n)=\frac{1}{2}\cdot [(\sum_{j=1}^{M}\bar{x_{j}}(n){)}^{2}-\sum_{j=1}^{M}\left|x_{j}(n)\right|]*f.$$

### 2.3. Minimum Variance Beamformer

The MV beamformer calculates adaptive weights through a constraint process that minimizes the power of the beamforming output [7]. We assume that the echo signal of a transmitting event is **x**(*n*); as in Equation (3), the output can be expressed as:

(8) $$y_{MV}[n]=w_{MV}^{H}[n]x[n],$$

where $w_{MV}$ is the MV weighting vector.

The constraint equation is as follows:

(9) $$\min\;w_{MV}^{H}R[n]w_{MV},subject\;to\;w_{MV}^{H}a=1,$$

where R[n] is the receiving array covariance matrix and a is the steering vector.

The solution of Equation (9) can be calculated by the Lagrange multiplier approach:

(10) $$w_{MV}=\frac{R^{-1}a}{a^{H}R^{-1}a}.$$

The covariance matrix, R[n], is usually estimated as:

(11) $$R[n]=E[x[n]x^{H}[n]].$$

Spatial smoothing and diagonal loading techniques are adopted to provide an invertible covariance matrix and a satisfactory robustness [26]. Then, the final covariance matrix can be expressed as:

(12) $$\hat{R}[n]=\frac{1}{M-L+1}\sum_{l=1}^{M-L+1}x_{l}(n)x_{l}^{H}(n)+\varepsilon \times I,$$

where $x_{l}$ is the *l*th subarray, *L* is the subarray length, ε represents the diagonal loading factor, and **I** is the identity matrix. ε is usually described as $\Delta \times \left(trace(\frac{1}{M-L+1}\sum_{l=1}^{M-L+1}x_{l}(n)x_{l}^{H}(n))/L\right)$, and the typical range of Δ is 0.01–0.2. Afterwards, R[n] would be replaced by $\hat{R}[n]$ in Equations (9) and (10).

### 2.4. Partial Generalized Sidelobe Canceler

The generalized sidelobe canceller (GSC) can be regarded as an equivalent form to implement the MV beamformer. It divides the weight vector into two parts: the upper constant and the lower adaptive vectors. In the whole structure, the beamforming output can be obtained by subtracting the noise and interference of the lower branch from desired signals of the upper branch [27,28].

In a single transmitting event, **x**[*n*] in Equation (3) represents the signal vector, and the output of the GSC can be expressed as:

(13) $$y_{GSC}[n]={\left(w_{q}-Bw_{a}[n]\right)}^{H}x[n].$$

Then, the constraint process of the MV in Equation (9) can be rewritten as:

(14) $$\min_{w_{a}[n]}{\left(w_{q}-Bw_{a}[n]\right)}^{H}\hat{R}[n](w_{q}-Bw_{a}[n]).$$

The solution of $w_{a}[n]$ is:

(15) $$w_{a}[n]={\left(B^{H}\hat{R}[n]B\right)}^{-1}B^{H}\hat{R}[n]w_{q},$$

where $w_{q}$ is the constant vector. B is a blocking matrix and $\hat{R}[n]$ represents the covariance matrix after spatial smoothing and diagonal loading.

The pGSC is implemented by combining the dimension reduction and the GSC [29]. A transformation matrix T can be put after the blocking matrix B, then the new partial blocking matrix would be ${\hat{B}}_{M\times NN}=B_{M\times M-1}T_{M-1\times NN}$. *NN* defines the degrees of freedom and B in Equations (13)–(15) would be replaced by $\hat{B}$ [17].

## 3. Methods

**Figure 1:**
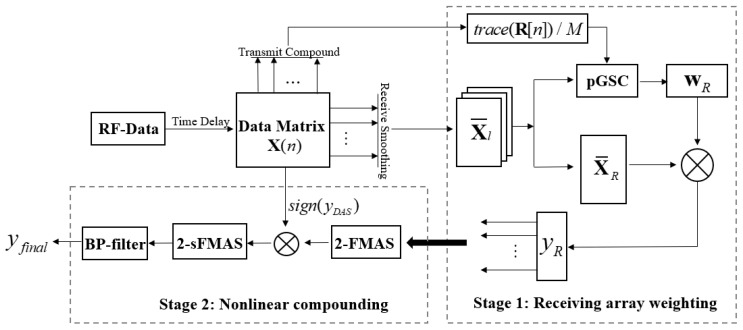
Overview of the proposed method.

The overview of the proposed method is shown in Figure 1. A high resolution and contrast ultrasound image can be obtained through a two-stage cascade structure.

### 3.1. Stage 1: Receiving Array Weighting

The data model of PWC imaging is as shown in Equation (1). Two dimensions of the matrix represent the receiving array and transmitting events, respectively. The submatrix smoothing process is implemented to smooth and de-correlate receiving array signals firstly. It is expressed as follows:

(16) $${\bar{X}}_{R}=\frac{1}{M-L+1}\sum_{l=1}^{M-L+1}{\bar{X}}_{l},$$

where *L* is the subarray length and *M* is the element number. The term ${\bar{X}}_{l}$ represents the *l*th overlapping submatrix:

(17) $${\bar{X}}_{l}=\left[\begin{array}{cccc} x_{1,l}(n) & x_{1,l+1}(n) & \cdots & x_{1,l+L-1}(n)\\ x_{2,l}(n) & x_{2,l+1}(n) & \cdots & x_{2,l+L-1}(n)\\ \vdots & \vdots & \ddots & \vdots \\ x_{N,l}(n) & x_{N,l+1}(n) & \cdots & x_{N,l+L-1}(n) \end{array}\right],$$

where $x_{i,l}(n)$ is used to represent the signal recorded in the original matrix in Equation (1).

Then, the multi-wave receiving covariance matrix R[n] is calculated by averaging over *N* transmissions [8]. The expression of R[n] is as follows:

(18) $$R[n]=\frac{1}{N(M-L+1)}\sum_{i=1}^{N}\sum_{l=1}^{M-L+1}x_{x_{i,l}}x_{x_{i,l}}^{H},$$

where $x_{x_{i,l}}={\left[x_{i,l}(n),x_{i,l+1}(n),\ldots ,x_{i,l+L-1}(n)\right]}^{T}$ stands for the *l*th receiving subarray vector of the *i*th transmitting event. Then the final receiving covariance matrix $\hat{R}[n]$ is obtained by adding a diagonal loading factor: $\hat{R}[n]=R[n]+\Delta \times (trace[R(n)]/L)\times I$. The term Δ is between 0.01 and 0.2 and I is the identity matrix.

The blocking matrix B with size L×(L−1) aims to block off desired signals in the adaptive road. Thus, it must satisfy:

(19) $$B^{H}a=0,$$

where **a** is the steering vector of ones.

In this paper, B is set as follows [30]:

(20) $$B={\left[\begin{array}{cccccc} 1 & -1 & 0 & 0 & \cdots & 0\\ 0 & 1 & -1 & 0 & \cdots & 0\\ \vdots & \vdots & \vdots & \vdots & \cdots & 0\\ 0 & 0 & \cdots & 0 & 1 & -1 \end{array}\right]}_{(L-1)*L}^{T}.$$

The discrete cosine transform matrix has been researched to represent original signals with lower dimensions by maintaining more energy [16]. It is adopted as the dimension reduction matrix T:

(21) $$T_{p,q}=\{\begin{array}{c} \frac{1}{\sqrt{M}}\;\;\;p=0,\;0\leq q\leq L-2\\ \sqrt{\frac{2}{M}}\cos\frac{\pi \left(q+\frac{1}{2}\right)p}{M}\;1\leq p\leq NN-1,\;0\leq q\leq L-2 \end{array}.$$

By placing T after B, the partial blocking matrix $\hat{B}$ is generated: ${\hat{B}}_{L\times NN}=B_{L\times L-1}T_{L-1\times NN}$.

Afterwards, Equation (10) is rewritten as:

(22) $$w_{a}[n]={\left({\hat{B}}^{H}\hat{R}[n]\hat{B}\right)}^{-1}{\hat{B}}^{H}\hat{R}[n]w_{q}.$$

The mathematical expansion [17] for the further calculation reduction is also implemented as follows:

(23) $$\begin{array}{ll} {\hat{B}}^{H}\hat{R}[n]\hat{B} & ={\hat{B}}^{H}\left(\frac{1}{N(M-L+1)}\sum_{i=1}^{N}\sum_{l=1}^{M-L+1}x_{x_{i,l}}x_{x_{i,l}}^{H}+\Delta \times \left(trace\left(R[n]\right)/L\right)\right)B\\ & =\frac{1}{N(M-L+1)}\sum_{i=1}^{N}\sum_{l=1}^{M-L+1}\left({\hat{B}}^{H}x_{x_{i,l}}\right)\left(x_{x_{i,l}}^{H}\hat{B}\right)+\Delta \times \left(trace\left(R[n]\right)/L\right)\cdot {\hat{B}}^{H}\hat{B}\\ & =\frac{1}{N(M-L+1)}\sum_{i=1}^{N}\sum_{l=1}^{M-L+1}\left({\hat{B}}^{H}x_{x_{i,l}}\right){\left({\hat{B}}^{H}x_{x_{i,l}}\right)}^{H}+\Delta \times \left(trace\left(R[n]\right)/L\right)\cdot {\hat{B}}^{H}\hat{B} \end{array}$$

(24) $$\begin{array}{ll} {\hat{B}}^{H}\hat{R}[n]w_{q} & ={\hat{B}}^{H}\left(\frac{1}{N(M-L+1)}\sum_{i=1}^{N}\sum_{l=1}^{M-L+1}x_{x_{i,l}}x_{x_{i,l}}^{H}+\Delta \times (trace\left(R[n]\right)/L)\right)w_{q}\\ & =\frac{1}{N(M-L+1)}\sum_{i=1}^{N}\sum_{l=1}^{M-L+1}\left({\hat{B}}^{H}x_{x_{i,l}}\right)\left(x_{x_{i,l}}^{H}w_{q}\right)+\Delta \times (trace\left(R[n]\right)/L)\cdot {\hat{B}}^{H}w_{q} \end{array}$$

Then, we estimate trace(R[n]) by $\bar{x}[n]{\bar{x}}^{H}[n]$ to avoid additional calculations. $\bar{x}[n]$ is the average of $x_{i}[n]$ in Equation (1), which represents the compounding data vector of different transmitting events.

After $w_{a}$ is calculated, the weighting vector of the receiving array can be obtained:

(25) $$w_{R}=w_{q}-\hat{B}w_{a},$$

where $w_{q}$ is set to a hamming window vector.

Weighting the receiving aperture of ${\bar{X}}_{R}$ in Equation (18), receiving dimension beamforming results can be calculated as follows:

(26) $$y_{R}={\left[y_{1},y_{2},\ldots ,y_{N}\right]}^{T}={\bar{X}}_{R}\cdot w_{R}.$$

### 3.2. Stage 2: Nonlinear Compounding

After obtaining the beamforming result of each transmission as in Equation (26), we rescale each term by applying a signed square root operation:

(27) $$z_{i}=sign(y_{i})\cdot \sqrt{\left|y_{i}\right|}.$$

Then a second signed square root operation is implemented as follows:

(28) $$u_{i}=sign(z_{i})\cdot \sqrt{\left|z_{i}\right|}.$$

Afterwards, the MAS is used to replace the DAS algebra in Equation (7) and the second-order FMAS (2-FMAS) is defined as:

(29) $$\begin{array}{l} y_{2-FMAS}=\frac{1}{2}\cdot [({\sum_{i=1}^{N}z_{i})}^{2}-\sum_{i=1}^{N}\left|y_{i}\right|]\\ \;\;=\frac{1}{2}\cdot \left[{\left(\frac{1}{2}\cdot \left[{\left(\sum_{i=1}^{N}u_{i}\right)}^{2}-\sum_{i=1}^{N}\left|z_{i}\right|\right]\right)}^{2}-\sum_{i=1}^{N}\left|y_{i}\right|\right] \end{array}.$$

Finally, the sign of the traditional DAS result is used to make a correction to the output of 2-FMAS [31]. Combined with the BP filter, the beamforming result can be obtained as:

(30) $$y_{final}=sign(y_{DAS})\cdot \left|y_{2-FMAS}\right|*f.$$

## 4. Experiments and Results

### 4.1. Experimental Setup

The proposed beamforming method is evaluated using the PWC through simulations and experiments. All simulated data were acquired by Field II [32], based on the MATLAB (R2019b) platform. All phantom and in vivo data were obtained through the Verasonics (Vantage 128, Verasonics, Redmond, WA, USA), which is a standard commercial ultrasound machine that cannot be modified. It is intended to be used as a research laboratory tool to acquire, store, display and analyze data. It follows the security verifications: “IEC 61010-1 3rd Edition (2010) and EN 61010-1:2010 3rd Edition” and “UL 61010-1: 2012 and CAN/CSA-22.2 No. 61010-1-12”. Further information about this instrument can be found through this website: https://verasonics.com/wp-content/uploads/2019/04/Vantage-Systems-Brochure.pdf. The collected data is only used for the ultrasound imaging. The participant has acknowledged the purpose of our experiments. All experiments comply with the Helsinki principles. The participant’s life, health, privacy and dignity have been guaranteed.

For simulations, both point targets and anechoic cyst were adopted. The central frequency of the transducer was 5 MHz, and the array had 128 elements and 0.3 mm pitch. During the transmitting process, 49 beams steering from −12 to 12° with an interval of 0.5° were generated to scan the imaging area. The excitation pulse was a 2-cycle sinusoid wave at the central frequency. The sampling frequency was set to 40 MHz. Five point targets at the depth of 20, 30, 40, 50 and 60 mm were simulated to evaluate the imaging resolution. A circular anechoic cyst with a radius of 2.5 mm centered at (x, z) = (0 mm, 50 mm) was also simulated to evaluate the imaging contrast. There were 200,000 scatters distributing in the cyst phantom randomly.

In experiments, a L11-4v transducer (Verasonics, Redmond, WA, USA) was used to acquire the phantom and in vivo data on the Verasonics platform. The parameter settings of the probe and transmitting process were the same as with the simulations. It is worth mentioning that the sampling frequency was firstly set to 20 MHz, and then the data was resampled at 40 MHz. The phantom data contained both point and cyst data, which were acquired using a CIRS calibration phantom (Model 040GSE, Computerized Imaging Reference System Inc., Norfolk, VA, USA). In vivo data from a 28-year-old male contained carotid arteries, parotid gland and thyroid data. Throughout the experiment, the mechanical and thermal index were kept to a minimum according to the ALARA principle since we have not made any changes to the instrument. The whole process is safe, non-invasive and non-radiation to the human body and the participant has acknowledged this.

Results of different methods: DAS, FMAS, 2-FMAS, 2-sFMAS, pGSC and 2-sFMAS pGSC will be shown in Section 4.2. First four methods are compared to test the effectiveness of the proposed nonlinear compounding stage in further reducing the sidelobe level and increasing the contrast. The pGSC was performed to evaluate the performance on narrowing the main lobe width and improving the resolution of the receiving array weighting stage. Finally, the 2-sFMAS pGSC was compared with previous methods, aiming to verify that our proposed scheme can achieve better imaging results in terms of both the resolution and contrast.

Another experiment was conducted to evaluate the robustness to noise of different beamformers. The anechoic cyst data in simulations were used and white Gaussian noise with the signal-to-noise ratio (SNR) of 10 dB, 0 dB and −10 dB were added.

Some parameters used in the experiment are illustrated here. The freedom of dimension reduction *NN* was set to 2, and the subarray length *L* is then selected as *M* – *NN* + 1 [17]. The selection of *NN* will be further discussed in the following part of this paper. The diagonal loading factor parameter Δ=0.01 was also used. The F-number was set to 1.5 in all beamformers and all figures are presented with −70 dB dynamic range. The BP filter was implemented by a 0.5 tapered Tukey window with the bandwidth between 3 and 12 MHz.

Evaluation metrics are explained here. The full width of the main lobe (FWHM), which is equally defined as the main lobe width at −6 dB, is used to evaluate the imaging resolution. For the quantitative measurement of the imaging contrast, the contrast ratio (CR) and contrast-to-noise ratio (CNR) are adopted in anechoic cyst results [33]. These two metrics are defined as:

(31) $$\mathrm{CR}=20\left|\log_{10}(\mu_{i}/\mu_{o})\right|,$$

(32) $$\mathrm{CNR}=\frac{\left|\mu_{i}-\mu_{o}\right|}{\sqrt{\sigma_{i}^{2}+\sigma_{o}^{2}}},$$

where $\mu_{i}$ and $\mu_{o}$ are the mean intensity of signals inside and outside the cyst. $\sigma_{i}$ and $\sigma_{o}$ are the standard deviation of $\mu_{i}$ and $\mu_{o}$, respectively.

### 4.2. Simulated Study

#### 4.2.1. Point Targets

**Figure 2:**
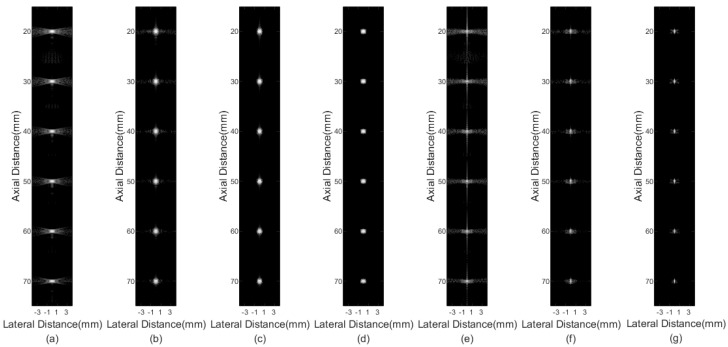
Simulated point targets imaging results: (**a**) DAS, (**b**) FMAS, (**c**) 2-FMAS, (**d**) 2-sFMAS, (**e**) pGSC, (**f**) FMAS pGSC, (**g**) 2-sFMAS pGSC. All figures are displayed within a −70 dB dynamic range. Abbreviations: DAS: delay and sum, FMAS: frame multiply and sum, 2-FMAS: second-order frame multiply and sum, 2-sFMAS: second-order signed frame multiply and sum, GSC: generalized sidelobe canceler, pGSC: partial generalized sidelobe canceler.

Figure 2 shows imaging results of different beamformers for point targets. Figure 2a is the result of the DAS, where serious sidelobes exist. In Figure 2b, although the FMAS shows its effectiveness in reducing the sidelobe level compared with the DAS, sidelobe signals are still visible. Figure 2c,d are results of the 2-FMAS and 2-sFMAS, respectively. The similar invisible level of the sidelobe is achieved, which proves the effectiveness of the proposed method for noise rejection. From Figure 2a–d, it can also be noted that the main lobe width of FMAS-based methods is similar to that of the DAS. Figure 2e shows that the pGSC has apparent advantages of narrower main lobe width, but sidelobe amplitudes are maintained high. Figure 2f is the result of the FMAS pGSC, narrower main lobe width and lower sidelobe levels are obtained compared with the pGSC in Figure 2e. The result of the 2-sFMAS pGSC in Figure 2g shows the best performance both on main lobe width and sidelobe levels, which indicates that the proposed method can bring high resolution and high contrast at the same time.

**Figure 3:**
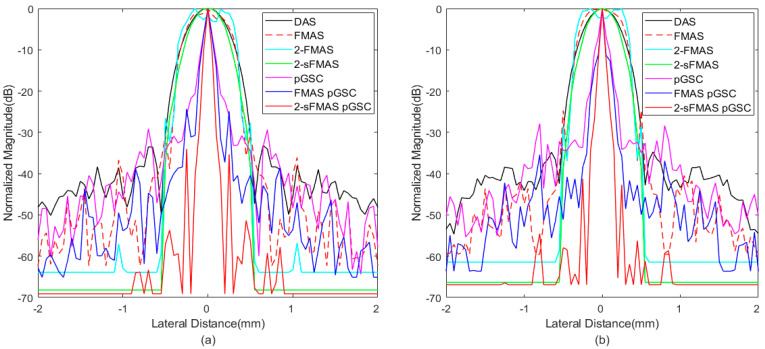
The lateral response at the depth of (**a**) *z* = 30 mm and (**b**) *z* = 50 mm.

In order to analyze results quantitatively, the lateral response of different beamformers at the depth of *z* = 30 mm and *z* = 50 mm is presented in Figure 3, and corresponding FWHM values are listed in Table 1. The proposed method achieves FWHM values of 0.08 mm/0.07 mm, which are much smaller than that of the conventional DAS method. From Figure 3, the best performance in narrowing the main lobe width and reducing sidelobe amplitudes of the proposed method is shown, especially for grating lobes. In addition, the 2-sFMAS shows a well-shaped curve without flatting at the top compared with the 2-FMAS.

#### 4.2.2. Anechoic Cyst

**Figure 4:**
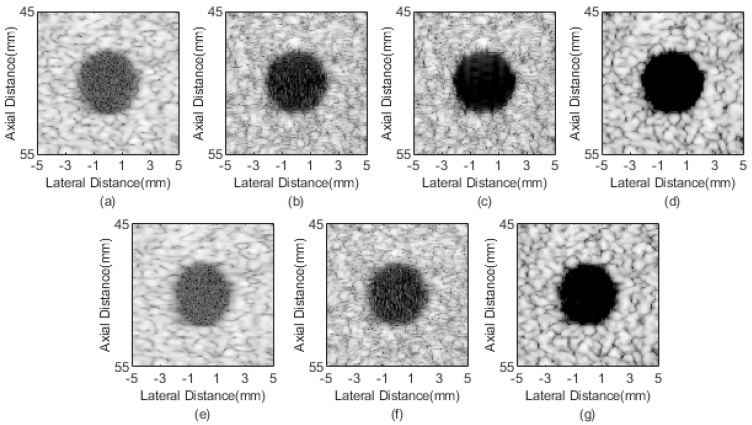
Simulated cysts imaging results: (**a**) DAS, (**b**) FMAS, (**c**) 2-FMAS, (**d**) 2-sFMAS, (**e**) pGSC, (**f**) FMAS pGSC, (**g**) 2-sFMAS pGSC. All figures are displayed within a −70 dB dynamic range.

Anechoic cyst results of different beamformers are shown in Figure 4. Obvious noise exists inside cysts in DAS and pGSC results, as shown in Figure 4a,e. The better performance in reducing noise are achieved by the FMAS and FMAS pGSC in Figure 4b,f, but room for improvement still exists. In comparison with the FMAS, the 2-FMAS shows effectiveness in further suppressing the internal noise, which indicates that the second-order operation is powerful in reducing off-axis scatter and sidelobes. Figure 4d is the sign correction result, a visible better speckle background and darker cyst can be seen in comparison with the 2-FMAS. Figure 4g shows a good performance similar to that of the 2-sFMAS. It means that the proposed method has superiority in the noise reduction and contrast improvement.

CRs and CNRs are calculated in Table 2. The values show that the FMAS achieves a higher CR at the cost of the CNR compared with the DAS and pGSC. The 2-FMAS further increases the CR, while the CNR decreases slightly in comparison with the FMAS method. The 2-sFMAS achieves the highest CR, and the CNR is better than those of the FMAS and 2-FMAS. The proposed 2-sFMAS pGSC achieves 112% and 121% higher CR than those of the DAS and pGSC, respectively, while the CNR is relatively reduced. It can also be noted that the proposed method performs slightly poor on the CR compared with only 2-sFMAS, but much better than only pGSC.

### 4.3. Experimental Study

#### 4.3.1. Point Target Phantom

**Figure 5:**
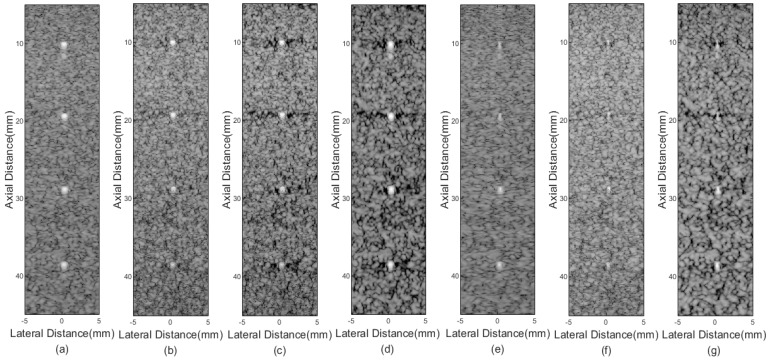
Experimental point targets phantom imaging results: (**a**) DAS, (**b**) FMAS, (**c**) 2-FMAS, (**d**) 2-sFMAS, (**e**) pGSC, (**f**) FMAS pGSC, (**g**) 2-sFMAS pGSC. All figures are displayed within a −70 dB dynamic range.

Results of different beamformers are shown in Figure 5. Similar to simulation results, pGSC-based methods show effectiveness in narrowing the main lobe as shown in Figure 5e–g, which indicate the higher spatial resolution compared with DAS and FMAS-based methods.

**Figure 6:**
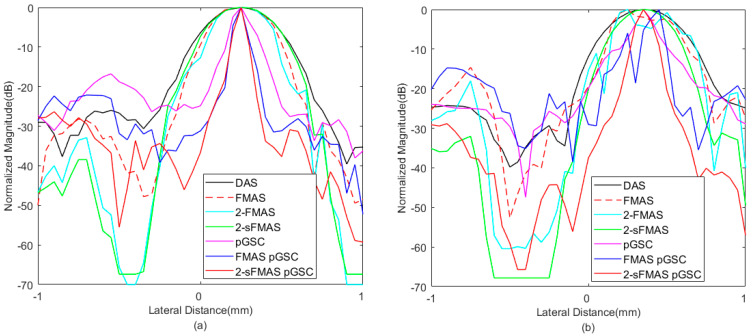
The lateral response at the depth of (**a**) *z* = 10 mm and (**b**) *z* = 29 mm.

Lateral response across the depth of *z* = 10 mm and *z* = 29 mm is shown in Figure 6, and the corresponding FWHM values are listed in Table 3. The proposed 2-sFMAS pGSC achieves 81.6% smaller FWHM than that of the DAS, which is consistent to simulated results. The pGSC-based methods achieve much smaller main lobe width compared with those FMAS-based methods.

#### 4.3.2. Cyst Phantom

**Figure 7:**
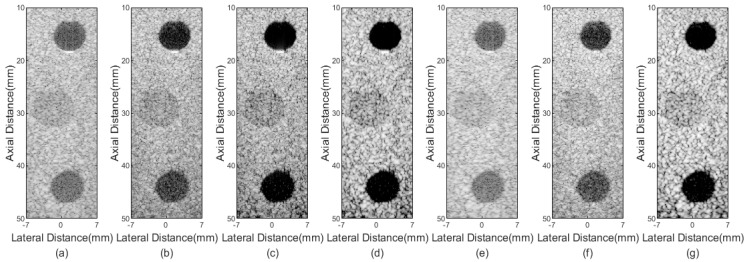
Experimental cysts phantom imaging results: (**a**) DAS, (**b**) FMAS, (**c**) 2-FMAS, (**d**) 2-sFMAS, (**e**) pGSC, (**f**) FMAS pGSC, and (**g**) 2-sFMAS pGSC. All figures are displayed within a −70 dB dynamic range.

In Figure 7, cysts phantom imaging results are presented. Figure 7c is the result of the 2-FMAS method, it shows less noise inside cysts and clearer anechoic cysts edges compared with the FMAS in Figure 7b. From Figure 7d,g, it can be seen that the 2-sFMAS and 2-sFMAS pGSC further reduce the internal noise and provide clearer edges of cysts, which are consistent with the simulated cyst results.

The statistical results of the CR and CNR are shown in Table 4. The 2-sFMAS gets the highest CR, which is 189% higher than the DAS, 126% higher than the FMAS. The proposed 2-sFMAS pGSC achieves a little smaller CR than that of 2-sFMAS, but it is still 183% higher than that of the DAS. It also can be seen that the FMAS-based methods result in a higher CR at the expense of the CNR, which is more obvious in the deeper anechoic cyst.

#### 4.3.3. In Vivo Study

**Figure 8:**
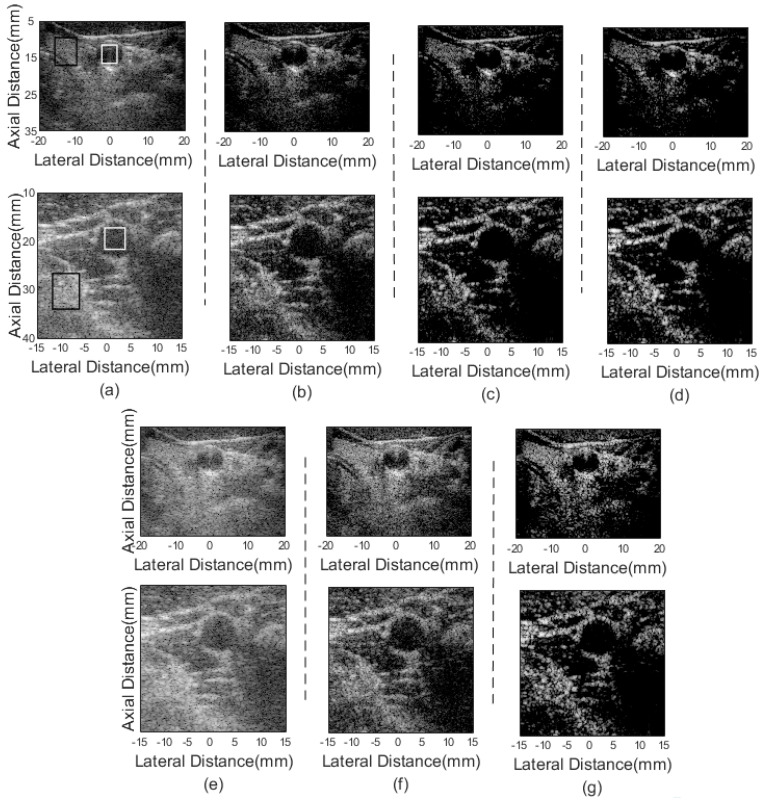
In vivo human carotid artery imaging results: (**a**) DAS, (**b**) FMAS, (**c**) 2-FMAS, (**d**) 2-sFMAS, (**e**) pGSC, (**f**) FMAS pGSC, and (**g**) 2-sFMAS pGSC. All figures are displayed within a −70 dB dynamic range.

Four sets of in vivo data are used to confirm the effectiveness of the proposed method. In Figure 8, the top row shows the right carotid artery imaging results and the bottom row presents the left carotid artery results. The anechoic circular structure in the figure is the cross-section of the blood vessel. The lower noise level inside the blood vessel and the more complete hyperechoic tissue preserved outside the blood vessel mean the better performance of the beamformer. Figure 8a,e present the DAS and pGSC results, respectively, which both show visible noise inside blood vessels. The imaging results of the 2-sFMAS and 2-sFMAS pGSC are shown in Figure 8d,g. They show the best performance in reducing noise inside vessels among all beamformers.

The quantitative assessment is also carried out, CRs and CNRs calculated between the rectangle region in white and black are listed in Table 5. The CR values of the DAS and pGSC are relatively small, while the CR values of the FMAS-based methods are significantly increased. The 2-sFMAS and the 2-sFMAS pGSC methods achieve higher CR values, which are 32.46/55.98 dB and 25.33/48.66 dB, respectively. However, it is worth noting that the value of the CNR has been sacrificed.

**Figure 9:**
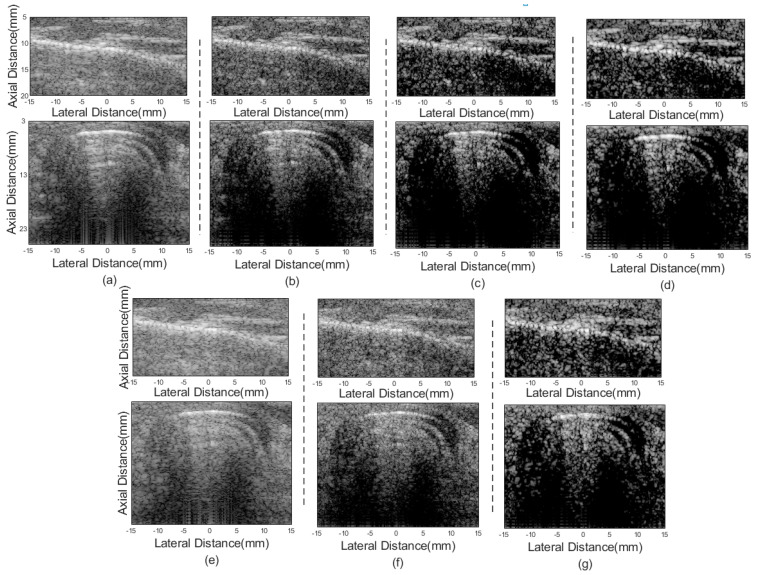
In vivo human parotid gland and thyroid imaging results: (**a**) DAS, (**b**) FMAS, (**c**) 2-FMAS, (**d**) 2-sFMAS, (**e**) pGSC, (**f**) FMAS pGSC, and (**g**) 2-sFMAS pGSC. All figures are displayed within a −70 dB dynamic range.

Another two datasets including human thyroid and parotid gland are also used to further evaluate the performance of different beamformers. The results are presented in Figure 9. The proposed method performs well on distinguishing the boundary between the parotid hyperechoic structure and the speckle background.

### 4.4. Robustness to Noise Evaluation

**Figure 10:**
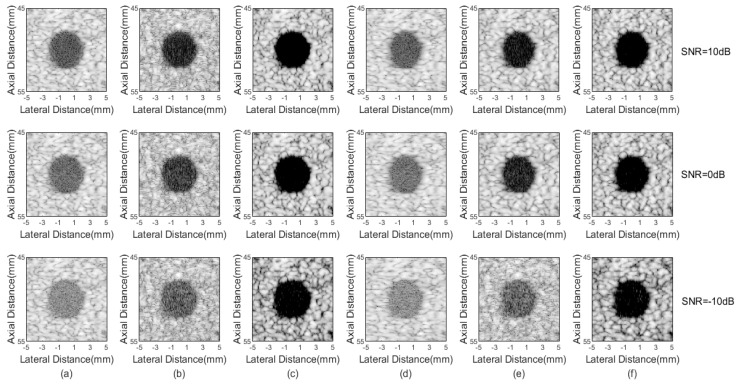
Results from simulated cyst phantom added Gaussian white noise with different levels: (**a**) DAS, (**b**) FMAS, (**c**) 2-sFMAS, (**d**) pGSC, (**e**) FMAS pGSC, (**f**) 2-sFMAS pGSC. All figures are displayed within a −70 dB dynamic range.

In ultrasound imaging system, the thermal noise usually exists in channels, which will affect the performance of the beamforming result. Therefore, the robustness against noise and interference experiment is necessary for evaluation. The data of the simulated anechoic cyst was used, and Gaussian white noise with SNR = 10, 0, and −10 dB were added to test imaging results of different beamformers. From Figure 10c,f, it can be seen that the proposed 2-sFMAS and 2-sFMAS pGSC methods perform best on the noise reduction inside the cyst under different noise levels. The edge of the cyst is clearer compared with other methods. Figure 10b,e show that the imaging results of the FMAS and FMAS pGSC are significantly degraded when the noise level is increased, although they are better than the DAS and pGSC. Statistical results are shown in Table 6. The proposed method obtained a 55.52 dB CR under the −10 dB noise level, which is still acceptable for tissue imaging. Overall, the proposed method is least affected by noise and has the best performance on the noise reduction.

## 5. Discussion

The results of simulations, experiments, and in vivo studies have been carried out. From results, the proposed method shows its effectiveness in narrowing the main lobe width and reducing sidelobe levels. Compared with the traditional DAS, the proposed method achieves a higher resolution and contrast, as shown in Figure 2a,g and Figure 4a,g, respectively. The higher resolution is owing to the receiving array weighting stage, as comparison of Figure 2a,e. In this stage, the adaptive weighting vector calculated through the pGSC structure plays an important role in reducing the main lobe width. Compared with the original MV method, the pGSC breaks the upper limitation of the subarray length during the smoothing process [17]. A larger subarray means that more effective aperture information is utilized, this also contributes to the high resolution. Meanwhile, the multi-wave covariance matrix is performed by integrating the information of all transmitting angles. This makes the covariance matrix more accurate and only one weighting vector is calculated. Combining the dimension reduction and the equation expansion step, the calculation amount remains relatively low.

The stage 2 shows its good performance in the noise rejection and contrast enhancement as shown in Figure 4 and Figure 7. In comparison with the FMAS, the 2-sFMAS shows better contrast as shown in Figure 4b,d. This is due to the replacement of the DAS by the MAS in the real-time reformulation of the FMAS, which achieves the secondary suppression of off-axis signals, thereby enhancing the contrast. In addition, the signal distortion and the flatten main lobe have been avoided through the sign correction process, which further improved the contrast performance. Furthermore, our method achieved better anti-noise performance when different levels of Gaussian white noise were added compared with the DAS and FMAS, as shown in Figure 10. The stage 2 contains additional square root and absolute value operations, but no more multiplications are added, so that it still maintains the ability of real-time implementation. Through the two stages, a high resolution and contrast image for the PWC is achieved. Furthermore, thanks to the dimension reduction, the formula expansion in the stage 1, and the real-time implementation in the stage 2, our proposed method maintains a low computational complexity.

For the selection of the dimension reduction freedom *NN*, it was set to 2 in this paper [16,17], and the results show that it performs well in all experiments. If *NN* is increased, more computations are required since the weight vector dimension and the number of subarrays are increased. With additional experimental explorations, we have found that increasing *NN* does not straightforwardly improve the imaging result, especially for the contrast. Meanwhile, *NN* = 1 also is not a good choice. It means that the degree of freedoms is reduced to one, which will limit the anti-noise performance of the proposed method [17]. The transducer frequency also affects imaging results. We have explored that the proposed method would be more advantageous on the imaging resolution at a lower frequency compared with other DAS and FMAS-based methods. It indicates that our method can make up for the poor imaging resolution of low frequency signals better.

The proposed method achieves a linear computational complexity. For one imaging point, an N*M data matrix and *NN* = 2 are used to implement the beamforming process. In the stage 1, the calculation amount mainly comes from the calculation of $w_{a}$. In Equation (17), R[n], ${\hat{B}}^{H}\hat{R}[n]\hat{B}$, ${\hat{B}}^{H}\hat{R}[n]w_{q}$, the inversion of ${\hat{B}}^{H}\hat{R}[n]\hat{B}$ and trace[R(n)] are needed to compute. The order of the computational complexity will be as high as $O(L^{2})$, if we calculate the multiplication of a matrix by a matrix directly. In detail, the calculation of R[n] requires $N\times L^{2}\times \left(M-L+1\right)=2NL^{2}$ multiplications, ${\hat{B}}^{H}\hat{R}[n]\hat{B}$ and ${\hat{B}}^{H}\hat{R}[n]w_{q}$ need about $NN\times L^{2}+\left(NN^{2}+NN\right)\times L=2L^{2}+5L$ operations in sum. Then, the total multiplications are about $2\left(N+1\right)L^{2}+5L$. However, by expanding ${\hat{B}}^{H}\hat{R}[n]\hat{B}$ and ${\hat{B}}^{H}\hat{R}[n]w_{q}$ in Equations (18) and (19), the complexity order can be decreased to O(L). To specify, we just need to compute ${\hat{B}}^{H}x_{x_{i,l}}$, $x_{x_{i,l}}^{H}w_{q}$ and trace[R(n)]. The complexity of ${\hat{B}}^{H}x_{x_{i,l}}$ and $x_{x_{i,l}}^{H}w_{q}$ are equal to NN×L and *L*, respectively. For trace[R(n)], it can be estimated by $\bar{x}[n]{\bar{x}}^{H}[n]$ with a complexity of *M*. The inversion of the 2×2 matrix can be easily obtained by exchanging the diagonal element positions with multiplication by determinant. Thus, the total calculation amount of $w_{a}$ for multiple transmitting events can be reduced to (NN+1)×L×(M−L+1)×N+M=6NL+M. In the stage 2, the square root, the absolute value and the sign of the DAS output are required and the process of multiplication in pairs is avoided. As a result, the complexity is expressed as *N*. In conclusion, the proposed method can be implemented by 6NL+M+N multiplications. It avoids the large calculation amount of the estimation and inversion of the covariance matrix through the dimension reduction and equation expansion steps. Thus, it could obtain faster imaging speed compared with JTR-based methods with the cubic computational complexity [8,12].

The advantages of the proposed method come from three aspects. First, a high resolution and contrast image can be achieved. Second, the robustness to noise can be further enhanced because of the good performance in suppressing noises. Third, the method is promising to be real-time implemented. However, there is still room for improvements. From the anechoic cyst simulation and experiment, it can be seen that the CNR value of the proposed method has been reduced. FMAS-based methods bring the measurement of the backscattered signal coherence into the beamforming chain, and thus achieve a better rejection of the uncorrelated noise. Although the proposed method further improves the performance on reducing the low-coherence noise, the over-suppression phenomenon is relatively more serious, resulting in the decrease of the CNR. Therefore, improving the CNR will be an aspect of our future work.

As for practical applications of the method, it is beneficial for the identification of tissues in medical clinical diagnosis. It could assist doctors to improve the accuracy of diagnosis of tumors, cysts and other diseased tissues. It also appears to be a promising tool for the cardiac ultrasound imaging [34]. The low computational complexity means the fast imaging speed. Combined with the GPU acceleration, it is more advantageous to be implemented in real time [35]. The high image quality also indicates that the number of frames can be saved, which is quite important to the imaging of rapid-moving tissues and organs. Furthermore, it may be beneficial for the ultrafast power doppler imaging, which is well-established to evaluate blood flows quantitatively with high frame rates [36,37]. The amplitude of the blood flow signal is much smaller than that of the tissue signal, and it is more susceptible to noise interference. Our method could provide good noise suppression and improve the effect of blood flow visualization. Other high frame rate strategies like 3D and 4D imaging, and transient elastography can also be further investigated with our beamforming method [1,6].

## 6. Conclusions

In this paper, a novel beamforming scheme is proposed to provide the high resolution and contrast image for the PWC. In our proposed method, a receiving weighting stage is implemented to beamform receiving array signals, which contributes to the high resolution. Then, an improved nonlinear compounding technique is proposed to compound the beamforming result of each transmitting event. This stage is beneficial for the noise suppression and contrast improvement. Both stages are implemented in low complexity forms. Thus, the proposed method achieves a linear computational complexity, which is more promising to meet the need of real-time imaging. Simulations, experiments and in vivo studies have been carried out to evaluate the effectiveness of our proposed method. The results confirm that our method is useful in improving the imaging quality. The robustness to the channel noise is also enhanced.

## Figures and Tables

**Table 1:** Full width of main lobe (FWHM) for simulated point targets at the depth of *z* = 30 mm and *z* = 50 mm.

Beamformer	FWHM (mm)
DAS	0.52/0.51
FMAS	0.55/0.60
2-FMAS	0.66/0.68
2-sFMAS	0.46/0.45
pGSC	0.10/0.09
FMAS pGSC	0.09/0.06
2-sFMAS pGSC	0.08/0.07

**Table 2:** Contrast ratio (CR) and contrast-to-noise ratio (CNR) for simulated cysts.

Beamformer	CR	CNR
DAS	33.85	1.83
FMAS	42.81	1.43
2-FMAS	51.57	1.39
2-sFMAS	79.77	1.53
pGSC	32.51	2.05
FMAS pGSC	39.29	1.53
2-sFMAS pGSC	71.72	1.44

**Table 3:** FWHM for the point phantom at the depth of *z* = 10 mm and *z* = 29 mm.

Beamformer	FWHM (mm)
DAS	0.49/0.53
FMAS	0.43/0.45
2-FMAS	0.40/0.45
2-sFMAS	0.48/0.42
pGSC	0.16/0.17
FMAS pGSC	0.09/0.14
2-sFMAS pGSC	0.09/0.15

**Table 4:** CR and CNR for experimental cysts.

Beamformer	CR	CNR
DAS	28.92/5.10/19.85	1.93/0.75/1.71
FMAS	42.35/5.42/25.35	1.65/0.68/1.34
2-FMAS	55.95/5.75/45.73	1.54/0.64/1.19
2-sFMAS	65.43/6.30/57.37	1.51/0.65/1.15
pGSC	27.53/4.03/19.91	2.06/0.62/1.76
FMAS pGSC	38.98/4.53/25.05	1.67/0.58/1.38
2-sFMAS pGSC	62.91/5.14/56.24	1.44/0.54/1.08

**Table 5:** CR and CNR for in vivo carotid artery.

Beamformer	CR	CNR
DAS	13.74/23.05	1.13/1.17
FMAS	20.23/30.68	1.17/0.80
2-FMAS	28.43/50.23	1.00/0.51
2-sFMAS	32.46/55.98	1.00/0.50
pGSC	11.78/22.01	1.20/1.31
FMAS pGSC	16.44/27.90	1.14/0.92
2-sFMAS pGSC	25.53/48.66	1.04/0.57

**Table 6:** CR and CNR for cyst phantom under different noise levels.

Beamformer	CR under 10/0/−10 dB Noise	CNR under 10/0/−10 dB Noise
DAS	32.56/27.46/18.74	1.82/1.79/1.66
FMAS	41.07/35.10/25.59	1.43/1.40/1.34
2-sFMAS	78.74/71.08/57.24	1.52/1.41/1.13
pGSC	31.33/26.31/18.53	2.04/1.99/1.81
FMAS pGSC	47.72/43.40/24.97	1.77/1.72/1.37
2-sFMAS pGSC	69.64/65.39/55.52	1.44/1.36/1.16

## Data Availability

The data presented in this study are available on request from the corresponding author. The data are not publicly available due to confidentiality reasons.

## Abbreviations

The following abbreviations are used in this manuscript:

2-DMAS	Second-order delay multiply and sum
2-FMAS	Second-order frame multiply and sum
2-sDMAS	Second-order signed delay multiply and sum
2-sFMAS	Second-order signed frame multiply and sum
Bp	Bandpass
CF	Coherence factor
CNR	Contrast-to-noise ratio
CR	Contrast ratio
DAS	Delay and sum
DC	Direct current
DMAS	Delay multiply and sum
FMAS	Frame multiply and sum
FWHM	Full width at half maximum
JTR	Joint transmitting-receiving
MAS	Multiply and sum
MTR	Mixed transmitting-receiving
MV	Minimum variance
pGSC	Partial generalized sidelobe canceler
PWC	Plane wave compounding
SNR	Signal-to-noise ratio
